# HIV-1 Tat complexes reveal subunit composition of active P-TEFb and stable association with 7SKsnRNP

**DOI:** 10.1186/1758-2652-13-S3-O5

**Published:** 2010-11-04

**Authors:** B Sobhian, N Laguette, A Yatim, M Nakamura, Y Levy, R Kiernan, M Benkirane

**Affiliations:** 1 Laboratoire de virologie moléculaire, Institut de Génétique Humaine, CNRS-UPR1142, Montpellier, France; 2 Laboratoire de régulation de l’expression des gènes, Institut de Génétique Humaine, CNRS-UPR1142, Montpellier, France; 3 INSERM U955, Faculté de Médecine de Créteil, Université Paris-Est, Hôpital Henri Mondor, Créteil, France

## Background

HIV-infected individuals harbour a latent reservoir, which is not accessible to current treatments and constitutes a major obstacle for virus eradication. Post-integration latency could result from transcriptional inhibition of the provirus. Efficient transcription of the viral genome requires recruitment of the positive transcription elongation factor b (P-TEFb) to the long terminal repeat by the viral protein, Tat. To better understand the regulation of viral transcription, we aimed at characterizing nuclear Tat-associated protein complexes.

## Methods

HeLa-S3 cells were stably transduced with C-terminally TAP-tagged Tat. Tat-associated complexes were purified from Dignam nuclear extracts by tandem affinity chromatography and interactors identified by tandem mass spectrometry. Biochemical and functional analysis using the siRNA approach were applied to understand the role of the newly identified Tat cofactors.

## Results

**Figure 1:**
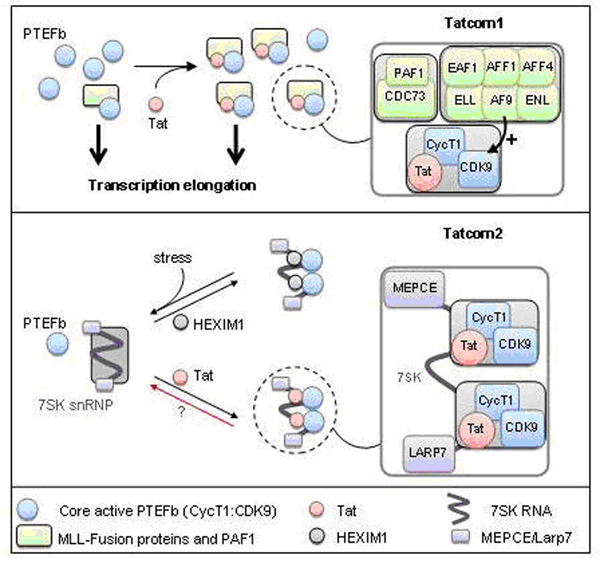
Model for HIV -1 Tatcom1 and Tatcom 2 assembly

Tat forms two distinct and stable complexes. Tatcom1 consists of core active P-TEFb, MLL-fusion partners involved in leukemia (AF9, AFF4, AFF1, ENL and ELL) and PAF1 complex. Importantly, Tatcom1 formation relies on P-TEFb, while optimal CDK9 CTD-kinase activity is AF9 dependent. MLL-fusion partners and PAF1 associate with elongating PTEFb as part of the regular cellular physiology. Tat increases the assembly or stability of this complex. Tatcom2 is composed of CDK9, Cyclin T1 and 7SK snRNP lacking HEXIM. Tat remodels 7SK snRNP by interacting directly with 7SKRNA, leading to the formation of a stress-resistant 7SK snRNP particle (Figure 1).

## Conclusions

We identified new factors required for Tat transactivation and important for P-TEFb function. Given the involvement of P-TEFb in HIV-1 transcriptional latency, they may represent new potential targets against HIV.

